# Dental Insurance Coverage, Dentist Visiting, and Oral Health Status among Asian Immigrant Women of Childbearing Age in Canada: A Comparative Study

**DOI:** 10.3390/healthcare11192666

**Published:** 2023-10-01

**Authors:** Qianqian Li, Meizhi Du, John C. Knight, Yanqing Yi, Qi Wang, Peizhong Peter Wang, Yun Zhu

**Affiliations:** 1 Division of Community Health and Humanities, Faculty of Medicine, Memorial University of Newfoundland, St. John’s, NL A1B 3V6, Canada; 2 Department of Epidemiology and Biostatistics, School of Public Health, Tianjin Medical University, Tianjin 300070, China; 3 Centre for New Immigrant Wellbeing, Markham, ON L3R 9V1, Canada; 4 Dalla Lana School of Public Health, University of Toronto, Toronto, ON M5T 3M7, Canada

**Keywords:** dental health, dentist visiting, dental health insurance coverage, self-perceived oral health, Asian women immigrants

## Abstract

Objectives: This study examined the dental insurance coverage, dentist visits, self-perceived oral health status, and dental problems among Asian immigrant women of childbearing age in contrast to Canadian women of childbearing age and non-Asian immigrant women of childbearing age. Potential barriers to dental care services among Asian immigrant women were explored. Methods: This analysis utilized data from the combined Canadian Community Health Survey from 2011 to 2014. The analytical sample consisted of 5737 females whose age was between 20 and 39 years. Multivariable logistic regression models assessed immigrant status and other factors in relation to the indicators of dental health (i.e., dental visit, self-perceived oral health, acute teeth issue, and teeth removed due to decay). Results: Amongst Asian women immigrants of childbearing age, there was a significantly lower frequency of dentist visits compared to non-immigrant counterparts (OR = 0.53; 95% CI: 0.37–0.76). The most commonly reported reason for not seeking dental care in the last three years was that the “respondent did not think it was necessary”. Relative to Canadian born women of same age bracket, Asian women of childbearing age reported fewer acute teeth issues (OR = 0.67; 95% CI: 0.49–0.91) and had a greater risk of tooth extracted due to tooth decay (OR = 3.31; 95% CI: 1.64–6.68). Furthermore, for Asian women immigrants, their major barriers to dental care included low household income (≤$39,999 vs. $40,000–$79,999 OR = 0.26) and a lack of dental insurance (no vs. yes OR = 0.33). Conclusions: Asian immigrant women showed lower utilization of dental services than non-immigrant women. A perceived lack of necessity, lower household income, and dental insurance coverage were major barriers to professional dental usage for most Asian immigrants of childbearing age.

## 1. Introduction

Compared with their male partners, immigrant women face heightened vulnerability to dental health issues due to factors such as lower wages, less job security, and decreased market participation in the Canadian labor market [1,2]. Several studies have highlighted that female immigrants are more likely to report dental problems and to use transnational dental care services over time [3,4,5,6]. Furthermore, women encounter increased oral health problems during pregnancy [7], although many of these conditions can be easily prevented or managed through timely and regular dental checkups [8]. Research suggests that some oral symptoms during pregnancy can have detrimental effects on pregnancy outcomes. For example, periodontitis has been positively associated with preterm birth and low birth weight, and high levels of cariogenic bacteria in mothers can contribute to dental caries in infants [9,10,11,12]. However, not only do Canadian government agencies lack authoritative guidance on dental care for pregnant women [13,14], but dentists, midwives, and pregnant women themselves have a deficiency in oral health knowledge and beliefs during the prenatal period [15]. As a result, patients, physicians, and dentists are often cautious and tend to avoid treatment during pregnancy [16]. Therefore, the preconception period has been understood as a more optimal time for treating prenatal periodontal disease. Nevertheless, only minimal research has reported the oral health problems among Asian immigrant women of childbearing age in Canada. This is the first quantitative examination of the oral health status and dental health issues among Asian immigrant women of childbearing age in Canada. The objectives of the present study included: (1) describing the oral health status and dentist visiting of Asian women immigrants at child baring age; (2) comparing the oral health status and dentist visiting of Asian immigrants with corresponding Canadian born female citizens and non-Asian female childbearing age immigrants; and (3) exploring factors that are associated with disparities in dental health and service utilization between Asian female childbearing age immigrants and other Canadian women of childbearing age.

## 2. Methods

### 2.1. Data Source

The Canadian Community Health Survey (CCHS) is a national population-based survey collecting detailed information on dentist visits, health status, and health risk factors for Canadian population. The CCHS interviews a sample of approximately 130,000 respondents and produces an annual master file combining two years of data. The CCHS conducted in 2011, 2012, 2013, and 2014 targeted Canadian citizens aged 12 or above who lived in 10 provinces or territories. Households were randomly selected, with the eligible respondents having a one-on-one interview with a computer-assisted personal interview (CAPI). The CCHS used a stratified three-stage sampling scheme in each geographic area in 10 provinces. In the initial stage, the sampling unit is a compact geographical area referred to as a cluster. In the subsequent stage, the sampling unit becomes the dwelling, and in the third stage, the sampling unit is the individual person. In order to be representative of the covered population, survey weights provided by Statistics Canada were used in calculations and statistical analysis [17]. In this cross-sectional study, the CCHS 2011–2014 master file data were analyzed through Statistics Canada’s Research Data Centers (RDC) Program at Memorial University.

The respondents provided self-reported information on their age, marital status, income, education, smoking status, alcohol consumption, immigrant status, dental insurance coverage, and diabetes status. To address any non-reported data, imputation and corrective actions were employed during the initial data collection process of the CCHS, aiming to minimize non-sampling errors [17,18]. Consequently, our dataset did not contain any missing values.

In the current study, childbearing age women were defined as females whose age was between 20 and 39 years. The CCHS questionnaire did not specifically collect information on “pregnancy status,” and thus our research did not selectively exclude either pregnant or non-pregnant subjects. The final analytical sample comprised 5737 women of childbearing age, ranging from 20 to 39 years old.

### 2.2. Outcome Variables

The first outcome focused on dental health status, which was assessed from two dimensions. The first dimension involved the perception aspect, captured through self-perceived dental health status. The second dimension pertained to the experience aspect, measured by the presence of any dental symptoms within the last month and the extraction of teeth due to decay in the past 12 months.

The second outcome variable was dentist visits, represented by the last time the participant visited the dentist and the frequency of dentist visits. Relevant questions and options in the questionnaires are listed in Table S1.

### 2.3. Independent Variables

In the current study, the main independent variable of interest was immigrant status, which was divided into ‘Canadian-born residents’, ‘Non-Asian immigrants’, ‘Asian long-term immigrants’ (10-years or more), and ‘Asian recent immigrants’ (<10 years). Canadian-born residents were defined as individuals who were born as Canadian citizens. Asian immigrants were defined as individuals born outside of Canada, who did not hold Canadian citizenship, and had an immigration status during the study (work or refugee) period. The ethnic origins categorized as Asian immigrants in this study included the following groups: Korean, Filipino, Japanese, Chinese, East Indian, Pakistani, Sri Lankan, Cambodian, Indonesian, Laotian, Vietnamese, Arab, Afghan, or Iranian. Non-Asian immigrants referred to individuals who were born outside of Canada, were not Canadian citizens by birth, held an immigration status in Canada during the study or work period, and had a racial background categorized as other than Asian. Other independent variables included demographic (e.g., age, gender, official language, and marital status), socioeconomic status (e.g., household income, education attainment, employment status, and dental insurance), health status (e.g., diabetes), and lifestyle factors (e.g., alcohol consumption, smoking, and tooth brushing frequency).

### 2.4. Statistical Analysis

Descriptive analyses compared the socioeconomic characteristics, demographic characteristics, lifestyle characteristics, and prevalence of diabetes between the childbearing age Canadian-born residents, non-Asian childbearing age female immigrants, and Asian childbearing age female immigrants and between native-born Canadians and subgroups of long-term and recent Asian immigrants. To account for the unequal probability of selection in CCHS, we used the rescaled weights for descriptive analysis. The rescaled weights were computed by dividing the original weight by the average original weights [19]. The percentages of people with different characteristics between Canadian-born residents and non-Asian immigrants and subgroups of recent and long-term Asian immigrants were compared with Chi-square. Multivariable logistic regression models were performed to examine immigrant status and other factors in relation to the indicators of dental health (i.e., dental visit, self-perceived oral health, acute teeth issue, and teeth removed due to decay). Statistical analyses were performed with SAS version 9.4 (SAS Institute Inc., Cary, NC, USA). *p* values below 0.05 and 0.01 were regarded as indicating statistical significance and high statistical significance, respectively.

## 3. Results

The respondents were 4066 Canadian-born women, 763 other immigrant women, and 908 Asian immigrant women (522 recent immigrants and 386 long-term immigrants) aged between 20 and 39 years in Canada (Table 1). In general, immigrant women (non-Asian immigrants, 62.00%; Asian immigrants, 66.12%) were married or living in common-law at a higher percentage than native-born women (47.78%). Asian immigrants (33.43%) or non-Asian female childbearing age immigrants (30.70%) had lower household income compared with their native-born counterparts (20.19%), but income differences shrunk with the length of immigration increase in Canada (22.66%). More than 86% of the female study population reporting brushing teeth no less than twice each day regardless of immigrant status. Most immigrants had a healthy lifestyle, 90.3% of Asian immigrants and 83.32% non-Asian immigrants were non-smokers, without diabetes, and non-heavy drinkers, while the proportion was lower in Canadian-born residents (66.38%).

Table S2 shows dental insurance coverage by immigrant status for women aged 20–39 years old. Canadian-born residents (73.77%) had significantly higher dental insurance coverage than immigrants (62.94% in non-Asian immigrants, 58.62% in Asian immigrants). There were no differences in the proportion of dental insurance coverage between Canadian-born residents (73.77%) and those of long-term Asian immigrants (68.33%) (*p* > 0.05). Employee-sponsored dental insurance accounted for the majority (>80%) of dental insurance coverage, and there were no differences in dental insurance type between non-immigrants and immigrants. Within the subgroup of Asian immigrants, long-term Asian immigrants (90.47%) showed a higher proportion than recent Asian immigrants (77.26%) in terms of employer-sponsored dental insurance.

In terms of access to dentist visits, the results presented in Table S3 indicate a noteworthy disparity between Canadian-born women and the female immigrant population. A higher proportion (77.67%) Canadian-born female citizens reported visiting a dentist within the past year, in contrast to 66.31% of non-Asian female immigrants and 59.38% of female Asian immigrants. Nevertheless, the disparity in dentist visits within the past year was no longer statistically significant between long-term Asian immigrants (70.46%) and native-born Canadian women (77.67%) (*p* > 0.05). Similarly, the findings indicate that a greater proportion of native-born Canadians (91.87%) had visited a dentist within the last three years, in contrast to 86.49% of non-Asian immigrants and an average of 86.16% among Asian immigrants, with these distinctions being statistically significant (*p* < 0.01).

The multivariable logistic regression analysis revealed additional insights. Specifically, Asian immigrant women (OR = 0.53; 95% CI: 0.37–0.76), particularly recent Asian immigrants (OR = 0.43; 95% CI: 0.28–0.68), had a decreased likelihood of visiting the dentist more than once per year compared to Canadian-born residents (Table 2). On the other hand, non-Asian immigrants (OR = 0.71; 95% CI: 0.50–1.00) and long-term Asian immigrants (OR = 0.71; 95% CI: 0.42–1.19) did not demonstrate a significant association (*p* > 0.05) with visiting the dentist more than once per year when compared to Canadian-born residents. Furthermore, after controlling for potential confounders, no substantial difference was observed regarding the occurrence of dental visits within the last three years between these groups and native-born Canadians.

Table 3 demonstrates potential risk factors related to low rates of dentist visits. For Canadian-born women and non-Asian immigrant women, those with post-secondary education, who did not smoke or drink heavily, had dental insurance, and who had self-preserved dental health as excellent, very good, or good were more likely to visit the dentist at least once per year, while low household income (no income–39,999 vs. 40,000–79,999 OR = 0.26; 95% CI 0.12–0.59) and lack of dental insurance (no vs. yes OR = 0.33; 95% CI 0.17–0.67) discouraged Asian immigrants from dentist visits.

Regarding the main reasons for not visiting the dentist within the last three years, there were clear differences between native-born Canadians and Asian immigrants (Table 4). Among the four main reasons identified, “other reasons” were the leading cause of not visiting the dentist for native-born Canadians (48.84%), with “haven’t get around to it” following as the second most common reason (20.76%), whereas the biggest reason for non-Asian immigrants (34.23%) and Asian immigrants (67.80%) was “did not think it is necessary” followed by other reasons.

Further, Table S4 shows the prevalence of self-reported dental health, dental symptoms, and teeth lost for women. Over 85% of the female population tended to rate their dental health as “excellent, very good, good”, and there was no significant difference among the three population groups. Referring to the prevalence of any dental symptoms and teeth lost due to decay, a significant difference showed up between non-immigrant women and immigrant women. Asian female immigrants of childbearing age had a much lower prevalence (42.96%) of any tooth symptoms compared with Canadian-born residents (53.42%). After controlling for length of residence in Canada, both recent (42.78%) and long-term Asian female immigrants (43.20%) reported lower rates of any tooth symptoms than native-born women. However, there were no significant differences between non-Asian female immigrants and native-born Canadian women. Interestingly, when it came to teeth removed due to decay, Asian female immigrants of childbearing age had more tooth loss (6.08%) than the non-Asian women (3.40%) and native-born women (2.12%) in Canada.

Multivariate logistic regression analyses indicated no significant disparities (*p* > 0.05) in self-reported dental health between Asian immigrant women and Canadian-born women (OR = 1.21; 95% CI: 0.69–2.11). Nevertheless, notable distinctions were observed in dental symptoms and tooth loss between the two groups (Table 5). Asian immigrants had a lower risk of experiencing dental symptoms (OR = 0.67; 95% CI: 0.49–0.91) and a higher rate of having teeth removed due to decay in the past 1 year (OR = 3.31; 95% CI: 1.64–6.68) compared to non-immigrants after the adjustment of all covariates.

Stratified multivariable logistic regression analyses were performed separately among population groups to predict the odds and identify the risk factors for self-reported dental health, teeth removed due to decay, and experiences with dental health symptoms (Table 6). For native-born women, a post-secondary degree (OR = 0.62; 95% CI: 0.45–0.85), high household income (low vs. high: OR = 1.46; 95% CI: 1.02–2.11), a healthy lifestyle (no smoke, no heavy drink, and no diabetes: OR = 0.47; 95% CI: 0.34–0.66), a higher frequency of dental visits (<1/years and never vs. ≥1/year: OR = 3.97; 95% CI: 2.78–5.66), and brushing teeth two or more times per day (OR = 0.49; 95% CI: 0.34–0.70) were related to better self-perceived dental health, while these factors were not associated with self-perceived dental health status in Asian immigrant or non-Asian immigrant women, except for dentist visiting frequency for non-Asian immigrant women.

Referring to teeth removal due to decay, similar associations were found in all three groups of women (Table 6). Native-born women who had higher than a post-secondary degree, who live in with high-income families, did not smoke nor drink heavily, and did not have diabetes were less likely to report teeth lost due to decay. The occurrence of dental symptoms did not show significant associations with socioeconomic or lifestyle factors. Among Canadian-born women (OR = 0.62; 95% CI: 0.47–0.81) and non-Asian immigrant women (OR = 0.34; 95% CI: 0.12–1.00), those who brushed their teeth more than once per day were less likely to report any dental symptoms during the past month. Canadian-born women who did not frequently visit the dentist were more likely to report dental symptoms (OR = 1.33; 95% CI: 1.06–1.68).

## 4. Discussion

This is the first comprehensive study on the dental health status, attitudes, and misconceptions related to dentist-seeking behaviors in the Asian female population at their childbearing age in Canada. The low frequency of dentist visits by Asian immigrant women indicated that unmet dental health needs existed among Asian female immigrants at their childbearing age. The cause for unmet dental health needs may be due to perceived lack of necessity, lower household income, and a lack of dental insurance coverage. Asian female immigrants might be conscious of a lower rate of their dental health, but they did not possess an effective way of detecting dental symptoms. Consequently, these potential dental symptoms may further deteriorate into tooth removal due to decay. Our data furthered the understanding of barriers to dentist visits for Asian female childbearing age immigrants.

It is known that women are more susceptible than men to various oral health problems (gingival, periodontal), especially during pregnancy [20]. Maternal oral periodontal disease and dental caries not only affect a pregnant woman’s own oral health status, but may also increase her risk of other diseases, resulting in adverse birth outcomes, as well as transmitting infection to the offspring [9,21,22]. It is surprising that the topic of oral health and dentist visiting behavior for women at their childbearing age is noticeably silent, despite extensive literature focused on pregnant women’s dental health and their dental care-seeking behaviors. In contrast to the broader population aged 12 and above examined in a previous study by our team, this research as an outgrowth focuses specifically on women of childbearing age [23]. This demographic encompasses women aged 20 to 39 years. The intention behind this selection is to precisely delve into the oral health challenges faced by Asian women immigrants of childbearing age. The findings of this study are expected to shed light on how to improve the health of Asian women immigrants prior to their first or subsequent pregnancy.

Results from this study are consistent with most others in pregnant women, which demonstrate that both periodontal disease and caries are highly prevalent, particularly among low-income women and members of racial and ethnic minority groups [9]. In particular, dental insurance coverage is an important predictor of dentist visits, with publicly insured adults experiencing higher levels of oral diseases but less likely to access dental services. According to one study, because the public dental health programs for adults are limited in availability and accessibility, low-income pregnant women still face great difficulty in obtaining dental services [24].

Multiple studies emphasize the importance of dental care during pregnancy despite concerns about risks [25,26]. Pregnant women delay treatment, and both obstetricians and dentists are cautious about dental procedures during pregnancy [25,27,28,29]. Thus, proactive dental care for women of childbearing age is crucial. However, this study revealed unoptimistic results among female Asian immigrants at their childbearing age. With respect to dental care usage, our study reveals that evident unmet dental care requirements persist among female Asian immigrants of childbearing age when compared to the broader Canadian population.

In certain high-income countries, including the United Kingdom and Australia, migrants from developing nations are also identified as being at risk for suboptimal oral health [30,31]. This trend was evident in our previous research, where Asian migrants frequently assessed their dental health as “fair” or “poor” [23]. However, this study reveals a more positive perspective regarding the dental health status of female Asian immigrants in their childbearing years. Moreover, our earlier investigation showed no significant difference of the dental symptoms’ occurrence between the whole Asian immigrants with Canadian-born residents. This study reveals a lower risk of experiencing dental symptoms among Asian female immigrants [23]. The findings from this study revealed that Asian immigrant women possessed equivalently good self-perceived health and a significantly lower prevalence of dental symptoms among the three groups. Among numerous risk factors which affected dental health status, marital status and age were two factors that significantly correlated with Asian immigrants’ prevalence of dental symptoms [23]. However, the results in this study did not show any such factors correlating with prevalence of dental symptoms in female Asian immigrants of child-bearing age. This phenomenon may be due to the fact that Asian women are more concerned about their body and appearance, including oral appearance, than Asian men [32,33]. However, the concern is that although our results demonstrated that Asian female immigrants hold certain good dental health status (lower prevalence of dental symptoms), they showed a significantly higher rate of teeth removed due to decay compared to the rest of the groups. This higher rate of teeth removed could be explained by the Asian concept of teeth lost and periodontal disease. Previous studies showed that Chinese people believed that total tooth loss was “normal”, and that tooth loss was seen as undesirable. Chinese women believed that tooth loss was caused by frequent childbirth. Some Chinese perceived tooth loss as an opportunity to avoid pain [34].

Another explanation for the higher rate of teeth removed may be insufficient dental care usage. Our study showed that apparent unmet dental care needs still existed in female Asian immigrants compared with the rest of Canadian population. This is consistent with previous studies that found female immigrants were more likely to have visited the dentist [3,29,35]. Research has already identified the progression of periodontal disease and gingival to tooth decay and dental plaque, from the beginning of swelling of tissues, pain, and tenderness to the late events in the pathogenesis of decay. The early stage of subsurface demineralization can only be detected microscopically [36,37]. However, Asian immigrants would only intend to access dental care in the presence of symptoms rather than preventive oral hygiene practices [38,39]. As a consequence, treatment was delayed, making prevention too late.

Our study supported a similar hypothesis that oral health beliefs and cultural values may affect care-seeking behaviors, and therefore indirectly lead to a consequence of high risk of teeth loss in Asian female immigrants. Future community health education strategies should fit with the cultural health beliefs of Asian immigrants, who are probably less likely to have knowledge about the value, function, and availability of existing professional dental care services.

This study has several limitations. Firstly, the use of the cross-sectional CCHS prevented the assessment of changes in health status over time and the establishment of causal relationships between risk factors, dental health status, and dentist visiting behavior. Secondly, due to confidentiality restrictions, the most recent CCHS annual master file available for analysis at the time of the research was the 2014 dataset. It is important to acknowledge that analyzing outdated CCHS data from 2011 to 2014 may introduce limitations, including the inaccurate representation of current health-related trends, the impact of changing demographics on applicability, and the influence of evolving immigration policies on health outcomes. Moreover, given the low subject numbers deemed confidential by Statistic Canada, “heavy drinking,” “smoking,” and “diabetes” were combined into a single variable, the “healthy lifestyle factor.” In future studies, exploring these potential confounders separately will be essential to understanding their individual effects.

## 5. Conclusions

Asian immigrant women had fewer dentist appointments than non-immigrant women, mainly due to perceived low necessity, lower income, and limited insurance. They reported equivalently good self-perceived health and lower dental symptoms than Canadian women of childbearing age and non-Asian female immigrants in that age group. Asian immigrant women also had a higher rate of tooth removal due to decay. Policymakers should consider the necessity of tailored outreach efforts, financial assistance, and broader insurance coverage to promote dental care accessibility for Asian immigrant women. Preventive education, cultural sensitivity in healthcare, and ongoing research are also important to address disparities in oral health. Future investigations should explore the causes behind the higher tooth extraction risk in childbearing-aged Asian immigrant women, particularly in light of the different cultural background.

## Figures and Tables

**Table 1:** Distribution of selected demographic, socioeconomic, and lifestyle characteristics, by immigrant status, women aged 20–39 years, Canada.

Characteristic	Canadian-Born Residents (*n* = 4066) (%) #	Non-Asian Immigrants (*n* = 763) (%) #	Asian Immigrants (*n* = 908) (%) #	Recent Asian Immigrant (*n* = 522) (%) #	Long-Term Asian Immigrants (*n* = 386) (%) #
Age					
20–29	53.39	36.01	40.37	43.52	36.11
30–39	46.61	63.99	59.63	56.48	63.89
Marital status					
Married/Common-law	47.78	62.00	66.12	69.08	62.11
Divorced/separate/widow/Single/Never married	52.22	38.00	33.88	30.92	37.89
Education					
Less than secondary	30.08	29.49	31.54	34.35 ^E^	27.74
Secondary and post-secondary	69.92	70.51	68.46	65.65	72.26
Household income					
no income–$39,999	20.19	30.70	33.43	42.88	20.66 ^E^
$40,000–$79,999	29.90	32.91	33.32	33.12	33.59
$80,000 or more	49.91	36.39	33.25	24.01	45.75
Smoking/Diabetes/Heavy drinker status					
No	66.38	83.32	90.30	91.19	89.09
Yes	33.63	16.68	9.70 ^E^	8.81	10.91 ^E^
Frequency of brush teeth					
≥2/day	88.02	92.53	88.65	90.17	86.59
<2/day or others ‡	11.98	7.47 ^E^	11.35 ^E^	9.83 ^E^	13.41 ^E^

Data source: Canadian Community Healthy Survey annual data 2011, 2012, 2013, 2014. (%) # All percentages are probability weighted. *n* = weighted sample size. ^E^ Coefficient of variation between 16.6% and 33.3%. Estimates are considered marginal and associated with high sampling variability. ‡ Others included don’t know/refusal/not stated.

**Table 2:** Odds ratios for the association between immigrant status and dentist visits among women aged 20–39 years.

	Unadjusted OR	Adjusted OR #	Adjusted OR ‡	Adjusted OR §
**Visiting Dentist within the Last 3 Years (Yes)**				
Canadian-born residents †	1.00	1.00	1.00	1.00
Non-Asian immigrants	0.56 (0.38–0.83) **	0.55 (0.37–0.82) **	0.67 (0.43–1.03)	0.79 (0.49–1.26)
Asian immigrants	0.54 (0.34–0.85) **	0.53 (0.34–0.84) **	0.69 (0.42–1.14)	0.80 (0.48–1.34)
Recent Asian immigrants	0.50 (0.28–0.87) *	0.49 (0.28–0.86) *	0.73 (0.38–1.42)	0.86 (0.43–1.73)
Long-term Asian immigrants	0.61 (0.27–1.35)	0.59 (0.27–1.31)	0.62 (0.28–1.41)	0.72 (0.32–1.60)
**Visiting dentist more than once per year (Yes)**				
Canadian-born residents †	1.00	1.00	1.00	1.00
Non-Asian immigrants	0.56 (0.42–0.77) **	0.54 (0.40–0.73) **	0.62 (0.45–0.84) **	0.71 (0.50–1.00)
Asian immigrants	0.42 (0.30–0.59) **	0.40 (0.29–0.57) **	0.46 (0.33–0.65) **	0.53 (0.37–0.76) **
Recent Asian immigrants	0.30 (0.20–0.45) **	0.29 (0.19–0.44) **	0.38 (0.25–0.58) **	0.43 (0.28–0.68) **
Long-term Asian immigrants	0.68 (0.41–1.13)	0.66 (0.39–1.09)	0.62 (0.37–1.06)	0.71 (0.42–1.19)

Data source: Combined Canadian Community Healthy Survey annual data of 2011, 2012, 2013, and 2014. † Reference group. * Significantly different from Canadian-born residents (*p* < 0.05), using bootstrap. ** Highly significantly different from Canadian-born residents (*p* < 0.01), using bootstrap. Abbreviations: OR, odds ratio. # Adjusted for age (Two age groups: 20–29, 30–39). ‡ Adjusted for age, sex, marital status, education, household income, and diabetes status/smoking status/alcohol consumption. § Adjusted for all factors which include age, sex, marital status, education, household income, diabetes status/smoking status/alcohol consumption, self-perceived dental status, dental symptoms, and dental insurance coverage.

**Table 3:** Associations of dentist visiting behavior with demographic, socioeconomic, and health-related factors among women aged 20–39 years, stratified by immigrant status.

Characteristic	OR 95% Confidential Interval ‡		
	Visiting Dentist More Than Once per Year (Yes)		
	Canadian-Born Residents	Non-Asian Immigrants	Asian Immigrants
Education			
Less than secondary †	1.00	1.00	1.00
Post-secondary degree	1.56 (1.21–2.02) **	2.38 (1.23–4.62) *	1.90 (0.87–4.14)
Age			
30–40 †	1.00	1.00	1.00
20–29	1.02 (0.78–1.32)	0.977 (0.50–1.89)	0.99 (0.42–2.40)
Household income			
$40,000–$79,999 †	1.00	1.00	1.00
$80,000–more	2.16 (1.58–2.94) **	1.20 (0.56–2.55)	0.89 (0.41–1.93)
no income–$39,999	0.56 (0.41–0.77) **	0.32 (0.15–0.70) **	0.26 (0.12–0.59) **
Marital status			
Married/Common-law †	1.00	1.00	1.00
Single/Never married/Widowed/Separated/Divorced	1.23 (0.94–1.61)	0.81 (0.39–1.66)	1.87 (0.77–4.55)
Smoker/diabetes/heavy drinker			
Yes †	1.00	1.00	1.00
No	1.34 (1.07–1.67) *	1.297 (0.57–2.94)	0.83 (0.24–2.91)
Dental insurance coverage			
Yes †	1.00	1.00	1.00
no	0.31 (0.24–0.40) **	0.48 (0.25–0.89) *	0.33 (0.17–0.67) **
Dental symptoms			
Yes †	1.00	1.00	1.00
No	1.08 (0.85–1.37)	0.97 (0.51–1.87)	0.74 (0.39–1.39)
Self-preserved dental health			
Fair and poor †	1.00	1.00	1.00
Excellent, very good, good	4.02 (2.81–5.76) **	3.25 (1.37–7.71) **	2.30 (0.77–6.89)
Immigrant years			
0–10 years †	NA	NA	1.00
11–high years	NA	NA	1.56 (0.83–2.94)

Data source: Combined Canadian Community Healthy Survey annual data of 2011, 2012, 2013, and 2014. † Reference group. * Significantly different from Canadian-born residents (*p* < 0.05), using bootstrap. ** Highly significant different from Canadian-born residents (*p* < 0.01), using bootstrap. ‡ Adjusted for all factors which include age, marital status, education, household income, diabetes status/smoking status/alcohol consumption, immigration length, self-perceived dental health, dental symptoms, and dental insurance coverage.

**Table 4:** Reasons for not visiting dentist within the last 3 years, women aged 20–39, by immigration status.

	Canadian-Born Residents (*n* = 330) (%) # †	Non-Asian Immigrants (*n* = 103) (%) #	Asian Immigrants (*n* = 126) (%) #
Haven’t get around to it §	20.76	16.91 ^F^	9.59 ^F^
Respondents did not think necessary §	13.96 ^E^	34.23 ^E^	67.80
Cost §	16.43 ^E^	15.62 ^F^	3.97 ^F^
Other reasons ‡ §	48.84	33.24 ^E^	18.65 ^F^

Data source: Combined Canadian Community Healthy Survey annual data of 2011, 2012, 2013, and 2014. † Reference group. (%) # All percentages are probability weighted. ^E^ Coefficient of variation between 16.6% and 33.3%. Estimates are considered marginal and associated with high sampling variability. ^F^ Coefficient of variation greater than 33.3%, estimate suppressed. § Responses are not mutually exclusive. ‡ Included “Haven’t got around to it”, “dentist did not think necessary”, “personal/family responsibilities”, “not available when request dentist”, “not available in area”, “transportation problems”, “did not know where to go”, “fear”, “not specified”, and “other (not specified)”.

**Table 5:** Odds ratios for the association between immigrant status and selected dental health status indicators of women aged 20–39 years.

	Unadjusted OR	Adjusted OR #	Adjusted OR ‡	Adjusted OR §
Self-Perceived Teeth Health (Fair/Poor)				
Canadian-born residents †	1.00	1.00	1.00	1.00
Non-Asian immigrants	1.44 (0.97–2.14)	1.51 (1.01–2.25) *	1.53 (1.02–2.29) *	1.48 (0.96–2.27)
Asian immigrants	1.31 (0.81–2.13)	1.36 (0.84–2.20)	1.409 (0.83–2.39)	1.21 (0.69–2.11)
Recent Asian immigrants	1.31 (0.66–2.62)	1.35 (0.68–2.69)	1.277 (0.61–2.67)	1.07 (0.50–2.29)
Long-term Asian immigrants	1.32 (0.70–2.48)	1.38 (0.73–2.61)	1.615 (0.83–3.20)	1.45 (0.71–2.95)
Acute teeth issue (Yes)				
Canadian-born residents †	1.00	1.00	1.00	1.00
Non-Asian immigrants	0.85 (0.66–1.09)	0.858 (0.66–1.11)	0.87 (0.66–1.13)	0.88 (0.67–1.14)
Asian immigrants	0.66 (0.49–0.89) **	0.664 (0.49–0.90) **	0.67 (0.50–0.91) **	0.67 (0.49–0.91) **
Recent Asian immigrants	0.65 (0.42–1.00) *	0.657 (0.43–1.02)	0.66 (0.43–1.01)	0.66 (0.43–1.01)
Long-term Asian immigrants	0.66 (0.46–0.95) *	0.673 (0.47–0.97) *	0.69 (0.48–1.01)	0.68 (0.46–1.01)
Teeth removed due to decay in past 1 year (Yes)				
Canadian-born residents †	1.00	1.00	1.00	1.00
Non-Asian immigrants	1.62 (0.65–4.06)	1.55 (0.62–3.88)	1.70 (0.66–4.39)	1.72 (0.65–4.50)
Asian immigrants	2.99 (1.51–5.91) **	2.89 (1.48–5.64) **	3.31 (1.65–6.66) **	3.31 (1.64–6.68) **

Data source: Combined Canadian Community Healthy Survey annual data of 2011, 2012, 2013, and 2014. Abbreviations: OR = odds ratio † Reference group. * Significantly different from Canadian-born residents (*p* < 0.05), using bootstrap. ** Highly significant different from Canadian-born residences (*p* < 0.01), using bootstrap. # Adjusted for age (Two age groups: 20–29, 30–39). ‡ Adjusted for immigrant status, age, education, marriage, household income, smoking status/alcohol consumption/diabetes status. § Adjusted for all factors which include immigrant status, age, education, marriage, household income, smoking status/alcohol consumption/diabetes status, teeth brush, visiting dentist more than once per year, and dental insurance.

**Table 6:** Stratified logistic regression of select dental status and dental issues by immigrant status, of women aged 20–39 years, Canada.

Characteristic	OR 95% (Confidential Interval) §								
	Self-Perceived Dental Health (Fair/Poor)			Dental Symptoms within Past One Month (Yes)			Teeth Removed Due to Decay (Yes)		
	Canadian Born Residences	Non-Asian Immigrants	Asian Immigrants	Canadian Born Residences	Non-Asian Immigrants	Asian Immigrants	Canadian Born Residences	Non-Asian Immigrants	Asian Immigrants
Education									
Less than post-secondary †	1.00	1.00	1.00	1.00	1.00	1.00	1.00	1.00	1.00
Post-secondary degree	0.62 (0.45–0.85) **	1.63 (0.67–3.94)	0.35 (0.10–1.22)	0.81 (0.645–1.01)	1.06 (0.59–1.88)	0.61 (0.29–1.29)	0.33 (0.15–0.75) **	1.89 (0.07–49.81)	0.40 (0.06–2.53)
Age									
30–40 †	1.00	1.00	1.00	1.00	1.00	1.00	1.00	1.00	1.00
20–29	1.04 (0.74–1.45)	1.34 (0.58–3.09)	0.52 (0.15–1.79)	1.19 (0.96–1.48)	0.94 (0.53–1.65)	0.557 (0.25–1.25)	0.60 (0.30–1.21)	0.97 (0.13–7.42)	--
Household income									
$40,000–$79,999 †	1.00	1.00	1.00	1.00	1.00	1.00	1.00	1.00	1.00
$80,000–more	0.77 (0.49–1.21)	0.60 (0.12–2.94)	0.67 (0.26–1.75)	0.93 (0.75–1.15)	1.09 (0.57–2.07)	1.22 (0.65–2.31)	0.69 (0.22–2.16)	--	1.39 (0.10–19.04)
no income–$39,999	1.46 (1.02–2.11) *	2.24 (0.82–6.12)	0.33 (0.10–1.06)	1.09 (0.84–1.41)	0.52 (0.24–1.14)	0.93 (0.44–1.95)	2.38 (1.01–5.62) *	4.55 (0.30–68.05)	0.55 (0.01–66.84)
Marital status									
Married/Common-law †	1.00	1.00	1.00	1.00	1.00	1.00	1.00	1.00	1.00
Other status than marriage/common-law	1.00 (0.71–1.42)	0.54 (0.23–1.26)	1.35 (0.40–4.57)	0.95 (0.76–1.18)	0.81 (0.44–1.48)	1.21 (0.59–2.49)	0.828 (0.402–1.703)	0.93 (0.09–9.69)	0.63 (0.001–364.46)
Smoke, diabetes, drink status									
Yes †	1.00	1.00	1.00	1.00	1.00	1.00	1.00	1.00	1.00
No	0.47 (0.34–0.66) **	0.45 (0.17–1.19)	2.27 (<0.001–>999)	0.85 (0.70–1.03)	1.19 (0.61–2.32)	0.60 (0.20–1.80)	0.44 (0.20–0.95) *	0.25 (0.02–3.39)	--
Teeth brush									
<2 a day	1.00	1.00	1.00	1.00	1.00	1.00	1.00	1.00	1.00
≥2 a day	0.49 (0.34–0.70) **	0.28 (0.08–1.00)	0.45 (0.10–1.95)	0.62 (0.47–0.81) **	0.34 (0.12–1.00) *	1.82 (0.49–6.79)	1.01 (0.49–2.11)	--	--
Dental insurance coverage									
Yes	1.00	1.00	1.00	1.00	1.00	1.00	1.00	1.00	1.00
No	1.17 (0.80–1.72)	0.87 (0.35–2.21)	0.69 (0.28–1.72)	0.86 (0.70–1.06)	1.28 (0.72–2.30)	0.91 (0.48–1.74)	1.09 (0.53–2.25)	1.55 (0.12–20.77)	0.39 (0.02–10.19)
Frequency of visiting dentist									
≥1/year	1.00	1.00	1.00	1.00	1.00	1.00	1.00	1.00	1.00
<1/years and never	3.97 (2.78–5.66) **	3.16 (1.44–6.92) **	1.69 (0.54–5.29)	1.33 (1.06–1.68) *	1.18 (0.65–2.13)	1.00 (0.53–1.88)	1.20 (0.54–2.68)	1.037 (0.20–5.37)	1.67 (0.20–14.01)
Immigrant years									
0–10 years	NA	NA	1.00	NA	NA	1.00	NA	NA	--
11–high years	NA	NA	0.90 (0.36–2.25)	NA	NA	0.93 (0.52–1.69)	NA	NA	--

Data source: Combined Canadian Community Healthy Survey annual data of 2011, 2012, 2013, and 2014. Abbreviations: OR = odds ratio. -- Not applicable due to the low number of subjects deemed confidential, which was not disclosed by the Research Data Center. † Reference group. * Significantly different from Canadian-born residents (*p* < 0.05), using bootstrap. ** Highly significant different from Canadian-born residents (*p* < 0.01), using bootstrap. § Adjusted for all factors which include immigrant status, age, education, marriage, smoking status/alcohol consumption/diabetes status, visiting dentist more than once per year, teeth brush frequency, dental insurance coverage. and dental insurance coverage. NA: Not applicable. Immigration years were not relevant for Canadian-born residents, and they were omitted for non-Asian immigrants due to their secondary focus in the study.

## Data Availability

The datasets generated by the survey research during and/or analyzed during the current study are available in the RDC center of Memorial University. The corresponding public use CCHS Microdata File could be accessed through the library of Memorial University: https://sda-artsci-utoronto-ca.qe2a-proxy.mun.ca/legacy_sda/html/cchs.htm (accessed on 30 September 2023). The dataset creation plan and analytics codes utilized for the purpose of this study are available from the authors of this manuscript upon request.

## Supplementary Materials

The following supporting information can be downloaded at: https://www.mdpi.com/article/10.3390/healthcare11192666/s1, Table S1: Questions and options in the questionnaire regarding self-perceived dental health status, dental symptoms during the last month, teeth removed due to decay in the past 12 months, the last time the participant visited the dentist, and the frequency of dentist visits; Table S2: Rates of dental insurance coverage in women aged 20–39 years, by immigrant status; Table S3: Rate of last time visiting dentist and dentist visiting behavior per year in women aged 20–39, by immigrant status; Table S4: Prevalence of self-perceived dental health, dental symptoms, and teeth loss of women aged 20–39, by immigrant status.
