# Supplementary material for: Dental Insurance Coverage, Dentist Visiting, and Oral Health Status among Asian Immigrant Women of Childbearing Age in Canada: A Comparative Study
Source: Healthcare (Basel). 2023 Oct 1;11(19):2666. doi: 10.3390/healthcare11192666 (PMC10572324; doi:10.3390/healthcare11192666)
Supplement: Supplementary file 1 [file healthcare-11-02666-s001.zip › healthcare-2588190-supplementary.pdf]

Table S1: Questions and options in the questionnaire regarding self-perceived dental health status, dental symptoms during the last month, teeth removed due to decay in the past 12-months, the last time the participant visited the dentist, and the frequency of dentist visits.

Questionnaire from CCHS	Answers	Categorized outcomes
"In general, would you say the health of your teeth and mouth?"	"Excellent" or "Very good" or "Good"	good oral health status
	"Fair" or "Poor"	poor oral health status
"In the past month, have you had: a toothache?" or "In the past month, were your teeth: sensitive to hot or cold food or drinks?" or "In the past month, have you had: pain in or around the jaw joints?" or "In the past month, have you had: other pain in the mouth or face?" or "In the past month, have you had: bleeding gums?"	Either question was answered "yes" by a respondent	Had dental symptoms during past one month
	All questions were answered "No" by a respondent	Do not had dental symptoms during past one month
"In the past 12 months, have you had any teeth removed by a dentist?"	"Yes"	Had teeth removed due to decay in past one year
	"No"	Did not have teeth removed due to decay in past one year
"When was the last time that you went to a dentist?"	"Less than 1 year ago" or "1 year to less than 2 years ago" or "1 year to less than 3 years ago"	Visiting dentist within the last 3 years (Yes)
	"3 year to less than 4 years ago" or "4 year to less than 5 years ago" or "5 or more years ago"	Visiting dentist within the last 3 years (No)
"Do you usually visit dentist"	"More than once a year for check-ups" or "about once a year for check-ups"	Visiting dentist more than once per year (Yes)
	"Less than once a year for check-ups" or "only for emergency care"	Visiting dentist more than once per year (No)

Table S2: Rates of dental insurance coverage in women aged 20-39 years, by immigrant status.

	Canadian born residences (n= 4066) (%)#†	Non-Asian immigrants (n= 763) (%)#	Asian immigrants (n= 908) (%)#	Recent Asian immigrants (n= 522) (%)#	Long-term Asian immigrants (n= 386) (%)#
Dental insurance coverage					
		P<0.01 **	*P*<0.01 **	*P*<0.01 **	
Yes	73.77	62.94	58.62	51.45	68.33
no	26.23	37.02	41.38	48.55	31.67
Persons who have dental insurance	Canadian born residences (n= 3000) (%)#†	Non-Asian immigrants (n= 480) (%)#	Asian immigrants (n=532) (%)#	Recent Asian immigrants (n=268) (%)#	Long term Asian immigrants (n= 264) (%)#
Employer-sponsored dental insurance					
					P<0.05 *
Yes	83.45	80.66	83.81	77.26	90.47
No/ Other‡	16.55	19.34	16.19	21.96 E	9.19 E

Data source: Canadian Community Healthy Survey annual data 2011, 2012, 2013, 2014
(%) # All percentages are probability weighted
n=weighted sample size
*Significant different from Canadian born residences ($p<0.05$), using bootstrap
**Highly significant different from Canadian born residences ($p<0.01$), using bootstrap
E Coefficient of variation between 16.6% and 33.3%. Estimates are considered marginal and associated with high sampling variability.
‡Others included don't know/ refusal/not stated

Table S3: Rate of last time visiting dentist and dentist visiting behavior per year in women aged 20-39, by immigrant status.

	Canadian born female residences (n= 4066) (%)#†	Non-Asian female immigrants (n=763) (%)#	Asian female immigrant (n=908) (%)#	Recent Asian female immigrant (n=522) (%)#	Long-term Asian female immigrant (n=386) (%)#
Visiting dentist within the last 3 years					
		P<0.01**	*P*<0.01**	*P*<0.05*	
Yes	91.87	86.49	86.16	85.16	87.51
No and others‡	8.13	13.51[E]	13.84[E]	14.84[E]	12.49[F]
Visiting dentist more than once per year					
		P<0.01**	*P*<0.01**	*P*<0.01**	
Yes	77.67	66.31	59.38	51.19	70.46
No	22.33	33.69	40.62	48.81	29.54[E]

Data source: Canadian Community Healthy Survey annual data 2011,2012,2013,2014
(%) # All percentages are probability weighted
n=weighted sample size
†Reference group
*Significant different from Canadian born residences (*p*<0.05), using bootstrap
**Highly significant different from Canadian born residences (*p*<0.01), using bootstrap
E Coefficient of variation between 16.6% and 33.3%. Estimates are considered marginal and associated with high sampling variability.
F Coefficient of variation greater than 33.3%, estimate suppressed
‡Included don't know/ refusal/not stated

Table S4: Prevalence of self-perceived dental health, dental symptoms, and teeth loss of women aged 20-39, by immigrant status.

	Canadian born residences† (n= 4066) (%)#†	Non-Asian immigrants (n= 763) (%)#	Asian immigrants (n= 908) (%)#	Recent Asian immigrants (n=522) (%)#	Long-term Asian immigrants (n=386) (%)#
Self-perceived health					
Excellent/very good/good	89.41	85.43	86.54	86.55	86.53
Fair & poor	10.59	14.57^{E}	13.46^{E}	13.45^{E}	13.47^{E}
Teeth removed due to decay in past one year					
			P<0.01**		
Yes	2.12	3.40	6.08^{E}	-	-
No/not visit dentist	97.88	96.60	93.92	-	-
Dental symptoms in past month					
			P<0.01**	*P*<0.05*	*P*<0.05*
Yes$^{\&}$	53.42	49.21	42.96	42.78	43.20
No/ Other‡	46.58	50.79	57.04	57.22	56.80

Data source: Combined Canadian Community Healthy Survey annual data of 2011, 2012, 2013, and 2014.

(%) #All percentages are probability weighted.

†Reference group.

*Significantly different from Canadian born residences (*p*<0.05), using bootstrap

**Highly significant different from Canadian born residences (*p*<0.01), using bootstrap

- Not applicable due to the low number of subjects deemed confidential, which was not disclosed by the Research Data Center

E Coefficient of variation between 16.6% and 33.3%. Estimates are considered marginal and associated with high sampling variability.

& Include had a toothache, teeth sensitive to hot or cold, pain in jaw joints, pain in mouth or face, bleeding gum. Responses are not mutually exclusive.
